# A protocol for the systematic review and meta-analysis of thigmotactic behaviour in the open field test in rodent models associated with persistent pain

**DOI:** 10.1136/bmjos-2020-100135

**Published:** 2021-01-22

**Authors:** Xue Ying Zhang, Jan Vollert, Emily S Sena, Andrew SC Rice, Nadia Soliman

**Affiliations:** 1 Musculoskeletal, Imperial College London, London, UK; 2 Clinical Neurosciences, University of Edinburgh, Edinburgh, UK

**Keywords:** pain, animal models, systematic review, meta-research

## Abstract

**Objective:**

Thigmotaxis is an innate predator avoidance behaviour of rodents and is enhanced when animals are under stress. It is characterised by the preference of a rodent to seek shelter, rather than expose itself to the aversive open area. The behaviour has been proposed to be a measurable construct that can address the impact of pain on rodent behaviour. This systematic review will assess whether thigmotaxis can be influenced by experimental persistent pain and attenuated by pharmacological interventions in rodents.

**Search strategy:**

We will conduct search on three electronic databases to identify studies in which thigmotaxis was used as an outcome measure contextualised to a rodent model associated with persistent pain. All studies published until the date of the search will be considered.

**Screening and annotation:**

Two independent reviewers will screen studies based on the order of (1) titles and abstracts, and (2) full texts.

**Data management and reporting:**

For meta-analysis, we will extract thigmotactic behavioural data and calculate effect sizes. Effect sizes will be combined using a random-effects model. We will assess heterogeneity and identify sources of heterogeneity. A risk-of-bias assessment will be conducted to evaluate study quality. Publication bias will be assessed using funnel plots, Egger’s regression and trim-and-fill analysis. We will also extract stimulus-evoked limb withdrawal data to assess its correlation with thigmotaxis in the same animals. The evidence obtained will provide a comprehensive understanding of the strengths and limitations of using thigmotactic outcome measure in animal pain research so that future experimental designs can be optimised. We will follow the Preferred Reporting Items for Systematic Reviews and Meta-Analyses reporting guidelines and disseminate the review findings through publication and conference presentation.

**Strengths and limitations of this study**

- Search strings were optimised for each database to ensure all relevant studies to this review can be identified and selection bias is eliminated by having no date and language restriction during the search.
- Stimulus-evoked limb withdrawal data will be extracted with the primary outcome (thigmotaxis) when assessed in the same cohort of animals so that the correlation between these behavioural outcomes can be studied.
- To prevent the risk of relevant studies from being falsely excluded, we decided to retain broader inclusion criteria during the initial screening phase, but this also means more efforts will be required at the full-text screening phase.
- High levels of variability in experimental designs, conduct and reporting are likely to be observed between studies and may limit a meta-analysis.
- Animal studies which possess high or uncertain risk of bias are likely to compromise the accuracy of the results.

## Introduction

Chronic pain is the leading cause of disability and disease burden globally.^1 2^ Over the past few decades, promising novel drugs with encouraging preclinical results have failed to show efficacy in clinical trials.^3–6^ The paucity of novel analgesics and the disparity of findings between preclinical and clinical studies raises questions about the validity of current animal pain research, including the construct validity of the conventional behavioural outcome measures, that is whether the outcome measures are measuring the concept which they intend to measure.

We cannot directly measure pain in laboratory animals and we therefore are reliant on surrogate outcome measures. The most used outcome measures in animal pain research are stimulus-evoked limb withdrawal. However, there are several limitations to these outcome measures and researchers question their validity for pain research. First, stimulus-evoked limb withdrawal is an involuntary response triggered by spinal reflexes and is unable to address other pain-related mechanisms in the neuraxis.^6^ Second, rodents are prey species and therefore are inclined to hide signs of suffering to avoid predation during stimulus-evoked behavioural assessments. Additionally, rodents can learn that premature withdrawal is associated with less stimulation and human interaction.^7^ Thus, there is a risk of obtaining inaccurate results when these outcome measures are used in studies which investigate the efficacy of novel analgesic drugs. Third, they have limited clinical relevance as they only reflect sensory gain phenomena, exhibited by a subset of neuropathic pain patients, and when present these measures do not correlate with pain reports from patients’.^8^ Hyperpathia cannot be addressed by stimulus-evoked withdrawal as it is challenging to measure explosive pain responses in animals.^9^ These sensory hypersensitivities evoked by pain do not correlate with the pain experience reported by patients with chronic pain. For example, patients with neuropathic pain predominantly experience spontaneous pain and tend to have sensory perturbation associated with loss of function (ie, numbness and dysesthesia).^6 8 10^ Lastly, information regarding the affective and physical aspects of pain in animals cannot be addressed by stimulus-evoked limb withdrawal, factors often emphasised in clinical studies. These limitations demonstrate the need to identify and validate alternative outcome measures in effort to better reflect the clinical situation and improve the predictive validity of animal research.

Employing complex ethologically relevant behaviours as a type of non-evoked pain-related outcome measures was first proposed in 2007 by researchers from Imperial College London and Queen Mary University of London,^11^ and has since been employed in several research studies, some include Andrews *et al*,^12^ Parent *et al*,^13^ Morland *et al*^14^ and Shepherd *et al*.^15^ The advantage of complex ethologically relevant behaviours as opposed to the conventional stimulus-evoked limb withdrawal is that they can assess and provide information about other aspects of pain, such as how does pain have an impact on the physical function and the emotional state of an animal. It is important to note that these behaviours are not pain specific and can be affected by various perturbations. In order to contextualise them to pain, studies should show that changes in these behaviours caused by models relating to pain can be reversed by known analgesics.

Thigmotaxis is an innate predator avoidance behaviour of rodents. It is characterised by the preference of a rodent to seek shelter, rather than expose itself to the aversive open area. The behaviour is usually displayed when rodents are under stress and can also be enhanced by increasing light intensity in the open area.^16^ It is commonly used in animal research of psychiatric disorders, its use in the pain field is relatively novel. The behaviour can be assessed in various paradigms, for example, the open field test and the elevated plus maze test, and can be measured in various ways, which include comparing the time spent by animals in the aversive inner zone as opposed to the time spent in the sheltered peripheral wall area and number of entries into the inner zone. It is postulated that when rodents experience pain they are less likely to conduct potentially risky behaviours such as exploratory activity like food seeking.^17^ This could reflect the clinical observations where patients with chronic pain are often associated with exacerbated avoiding behaviours and anxiodepressive disorders.^18 19^ Empirical evidence has shown that increasing rodent thigmotactic behaviour correlates with several experimental models associated with chronic pain.^11 20–23^ It has also been shown that clinically approved analgesics such as gabapentin and morphine reduced thigmotactic behaviour in rodents with experimental persistent pain,^11 23 24^ suggesting this measurable construct is sensitive to clinically approved analgesics.

Using thigmotaxis as a pain-related behavioural outcome measure is an emerging area of animal pain research; therefore, we have taken the initiative to conduct a systematic review and meta-analysis to assess the strengths and limitations of the current experimental design of the open field test thereby guiding future study design optimisation. This systematic review will include studies that investigated changes in thigmotactic behaviour caused by any rodent models that are associated with persistent pain. We anticipate some of the studies will also have investigated the effect of pharmacological interventions on thigmotaxis and therefore we will also assess whether thigmotactic behaviour can be changed by pharmacological interventions. We will also extract stimulus-evoked limb withdrawal data of the same cohort of animals used in the open field test in order to assess the strength of correlation and whether they vary between disease models associated with persistent pain. The systematic review will also explore the impact of protocol variation on heterogeneity, evaluate internal validity and assess publication bias of animal studies in this field.

## Methods

This protocol was developed in accordance with the SYRCLE (Systematic Review Centre for Laboratory Animal Experimentation’s) protocol for systematic reviews of animal studies.^25^ The present protocol is registered on PROSPERO (CRD42020208044). To date, we have completed preliminary searches and piloted study selection to inform the development of the protocol. Formal screening has not begun.

### Research question

Does experimental persistent pain affect thigmotactic behaviour in rodents and can this behaviour be altered by administering pharmacological intervention(s)?

### Preliminary search and pilot study selection

Search terms were initially developed for three domains: laboratory rodent animals (the studied population), persistent pain (the condition of interest) and thigmotaxis (the outcome of interest). At the time of developing the search, we aimed for sensitivity over specificity, where we chose to use comprehensive animal search filters.^26 27^ However, the search led to the retrieval of ~16 000 studies after duplicates were removed which is a significant screening burden. Based on our understanding of the field, and expectation of a few hundred studies, we have further developed the search strategy with the aim of improving the specificity. The new search terms cover four domains: laboratory rodent animals (the population), persistent pain (the condition of interest), sensory phenotypes (pain-specific terms) and thigmotaxis (the outcome of interest) (table 1). The pain-specific terms were adapted from another systematic review of preclinical pain studies^28^ and a literature search identified the different terms used to describe thigmotaxis.

**Table 1:** The general search strings

Component	Terms
Laboratory rodent animals	Rodentia OR rodent OR rodents OR rat OR rats OR rattus OR norvegicus OR mouse OR mice OR murinae OR muridae OR murine OR mus OR musculus OR woodmouse OR apodemus
AND	
Thigmotaxis	Thigmotaxis OR thigmotactic OR open field OR anxiety OR fear
AND	
Pain	Spinal cord injury OR nerve injury OR nerve injuries OR nerve transection OR nerve ligation OR neuropathy OR peripheral neuropathy OR polyneuropathy OR neuropathic OR headache OR headache-like OR migraine OR migraine like OR arthritis OR osteoarthritis OR rheumatoid arthritis OR colitis
AND	
Sensory phenotypes	Pain OR hyperalgesia OR analgesia OR analgesic OR analgesics OR allodynia OR neuralgia OR hypersensitivity OR hyperalgesic OR hyperalgesia OR antinociception OR anti-nociception OR hypoalgesia OR hypoalgesic OR antihyperalgesia OR antihyperalgesia OR antihyperalgesic OR anti-hyperalgesic OR anti-allodynic OR antiallodynic OR anti-allodynia OR antiallodynia

Since thigmotaxis can be referred to in many ways, there is a potential risk of falsely excluding relevant studies when a novel term is used to describe the behaviour in titles and abstracts. Furthermore, information is not always reported in detail in abstracts of preclinical studies, therefore studies with abstracts that did not report information listed in the inclusion criteria are also likely to be falsely excluded. To determine whether title and abstract screening would lead to incorrectly excluding studies we conducted pilot screening. If studies were wrongly excluded, then we would consider full-text screening from the outset; otherwise, we would conduct the standard screening procedures. One hundred studies were randomly selected from different pages of the PubMed search results. The page numbers were determined by using a random number generator. The studies were equally divided into two groups, of which one was screened based on title and abstract and one based on full texts. Of the title and abstract screening group, 11 studies met the inclusion criteria, whereas only 2 studies were eligible in the full text screening group. The lower inclusion prevalence in the full-text screening group was reasonable because deeper study eligibility assessment was carried out in this group. Additionally, we performed full-text screening of the 39 studies which were initially excluded based on their titles and abstracts. We found that all the studies were correct exclusions (ie, not rodent nor persistent pain) so the pilot screening could not address the issue of false exclusion. Moving forward, we have decided to retain broader inclusion criteria during the title and abstract screening (ie, include studies that report pain-associated rodent behaviours in abstracts). This strategy of overinclusion of studies will prevent the risk of falsely omitting relevant studies, thereby retaining sensitivity during title and abstract screening. The eligibility of these studies will then be confirmed during full text screening.

### Bibliographic search

The systematic search for relevant literature will be conducted on the three electronic databases; PubMed/Medline, Embase and Web of Science. All studies published until the date of the search will be considered and there will be no language restriction. If studies are published in non-English languages, we will use the Imperial College London library translation service. Reference lists of the included studies will be manually searched and any studies matching the eligibility criteria will be included in the full text screening.

### Screening

The screening process will be completed by a minimum of two independent reviewers and any discrepancies will be resolved by a third reviewer. Studies will be screened in two phases: (1) titles and abstracts will be screened against the inclusion and exclusion criteria outline below; (2) full texts of eligible studies identified from the first screening phase will be further assessed for final inclusion.

### Inclusion and exclusion criteria

Studies will be screened according to the criteria outlined below:

- Study design—comparative studies that investigate change in thigmotactic behavioural outcomes in rodents with an experimental model associated with persistent pain and that has at least one appropriate control presented will be included. The control population is defined as a cohort of naïve animals (eg, healthy control) and/or a cohort of sham animals (eg, model control). A wild-type control is required if transgenic animals were used to study persistent pain.
- Animal model—any rodent models (in vivo) that may be associated with persistent pain will be included. This includes any pathological form (ie, induced chemically, surgically or genetically) of persistent (ie, developed over a period of hours, weeks or months) pain. Non-rodent studies, in vitro and ex vivo studies or studies of acute nociception will be excluded.
- Outcome measure—any thigmotactic behavioural metrics assessed during an open field test will be included. For example: distance travelled in inner zone, frequency entry to inner zone, frequency of corner entry, latency to inner zone entry, time spent in inner zone, time spent in corner.
- For interventional studies (including studies in which solely investigated the effect of pharmacological interventions on thigmotaxis and did not quantify the model effect on thigmotaxis)—a vehicle control is required when studies investigated the effect of pharmacological interventions on thigmotactic behaviour in rodents with experimental persistent pain. We will include any potentially analgesic pharmacological interventions that is administered prior to, after or in concurrence with the model induction.

- Other criteria—only include original research articles; abstract only publications or other publication types such as reviews, letters or editorial materials will be excluded.

## Data extraction

The data extraction will be conducted by two independent reviewers. Any discrepancies will be resolved by a third reviewer. We will collect the following information:

- Bibliographic details.
- Outcome data.
- Acclimatisation and animal husbandry.
- Animal model.
- Intervention.
- Characteristics of the open field paradigm.
- Reporting quality.
- Study quality.

Additionally, we will ascertain whether a study protocol, registered before the study began is available. This will be done by assessing whether authors have provided a statement regarding protocol registration. Protocol registration can help to increase research transparency and reduce the risk of reporting bias. By collecting this information, we can also assess whether there is an increasing trend of protocol registration within this field.

### Bibliographic details

We will extract names of first authors, year of publication and title.

### Outcome data

The primary outcomes are total distance travelled and any behavioural data that denote thigmotaxis during the open field test, which are:

- Distance travelled in inner zone.
- Frequency of entry to inner zone.
- Frequency of corner entry.
- Latency to inner zone entry.
- Time spent in inner zone.
- Time spent in corner.

The secondary outcome is mechanical (Randall-Selitto, von Frey), cold (acetone, cold plate, tail immersion) or thermal (Hargreaves test, hot plate, tail immersion, tail flick) evoked limb withdrawal assessed in the same cohort of animals used in the open field test.

We expect most studies to only report at a single time period. When multiple time periods are reported, we will extract the data at the time period of the maximum effect (ie, the time period at which there is the largest difference between treatment and control groups). The sample size of each cohort will be extracted and if sample size is given as a range, the most conservative estimate will be extracted (eg, n=4–8, 4 will be recorded). The mean and the variance (eg, SE of the mean, SD) of thigmotactic behavioural data will be extracted from tables, text or graphs. If the data are presented within a graph, we will manually extract the data using digital ruler software (eg, WebPlotDigitizer). If the nature of the error bar is not reported, we will regard them as standard errors of mean. We will only contact authors when key information is not reported (eg, sample size, outcome data). If the authors do not respond or are not able to provide the data, we will record the study as having missing data and it will be qualitatively analysed but will not be included in the meta-analysis.

In addition to collecting thigmotactic behavioural data, we will also extract study characteristics. The details of each are described below:

### Acclimatisation and animal husbandry

The following information will be extracted:

1. Time period of acclimatisation to housing environment following transportation.
2. Housing condition (ie, presence of other animal species and/or sex in the room, number of animals per cage, temperature, humidity, noise, vibration).
3. Cage condition (ie, reporting of cage size, cage floor condition).
4. Light–dark cycle.
5. Feeding regime (ie, type of diet).

### Animal model characteristics

The following characteristics will be extracted:

1. Species.
2. Strain.
3. Sex.
4. Animal supplier.
5. Age (at the start of experiments).
6. Weight (at the start of experiments).
7. Model of persistent pain.
8. Method of model induction (ie, surgical procedure, dose and route of pharmacological administration).
9. Perioperative analgesic(s) given before/during/after model induction.

### Intervention characteristics

For studies investigating the effect of pharmacological interventions on thigmotactic behaviour in rodent models of persistent pain, the following characteristics will be extracted:

1. Type of intervention.
2. Vehicle control.
3. Dosing regimen (ie, dose, timing, frequency of administration, duration of treatment).
4. Route of administration.
5. Time between treatment and model induction.
6. Time between treatment and behavioural assessment.

### Characteristics of the open field paradigm

1. Size and shape of the open field arena (ie, length, height, width, total area in cm^2^).
2. Size and shape of the inner zone (ie, length, width, total area in cm^2^).
3. Colour of the test arena wall.
4. Experimental environment (ie, light intensity, temperature, humidity, noise, vibration).
5. Location of where animal was placed at the start of the test (ie, in the centre or on the edge).
6. Method of measurement (ie, automated or manual).
7. Manufacturer of the recording camera and type of the analysis software.
8. Habituation time to test arena.
9. Assessment time.
10. Number of trials and the time separation between trials.
11. Time between the model induction and the first open field test.
12. Time between the model induction and the last open field test.
13. Was the test conducted in an isolated chamber? If not, whether other animals were present in the testing room? And, whether the investigator was present in the test room during the assessment?

### Characteristics of the nociceptive assessment

1. Type of assessment (ie, mechanical, thermal, cold).
2. Habituation time.
3. Method of assessment.
4. Time between the model induction and the first outcome assessment.
5. Time between the model induction and the last outcome assessment.

### Reporting guideline

Since the use of ethologically relevant behaviours as pain-related outcome measures is relatively novel, we will extract whether studies published from 2010 onwards were reported in accordance with a recognised reporting guideline, such as the ARRIVE (Animal Research: Reporting of In Vivo Experiments) Guidelines^29^ and the Landis 4 criteria.^30^ If so, the following information will then be extracted:

1. Specify the used reporting guideline.
2. Evidence of reporting in accordance with the chosen guideline (ie, a checklist).

### Study quality: risk-of-bias assessment

By adapting the CAMARADES (Collaborative Approach to Meta-Analysis and Review of Animal Data from Experimental Studies) checklist for study quality,^31^ the following items will be evaluated in order to assess the methodological quality of each study:

1. Reporting of random allocation.
2. Reporting of allocation concealment.
3. Reporting of blinded assessment of outcome.
4. Reporting of sample size calculation.
5. Reporting of animal exclusions (eg, reasons and number of excluded animals).

We will also assess whether studies provided statements regarding:

1. Potential conflict of interest.
2. Compliance with animal welfare regulations.

Reviewers will state whether each item is reported and provide a description of the method that the researchers used. We will use the SYRCLE risk-of-bias tool^32^ to rate each item separately: low risk (accepted methods and are adequately described), high risk (inappropriate methods that do not efficiently mitigate bias) or unclear risk (details of methods insufficiently reported).

## Data synthesis

Meta-analysis will only be conducted if there is ≥10 independent comparisons. If not, a descriptive summary will be presented. Studies with insufficient data (eg, missing sample size, etc) will be excluded from the meta-analysis but will still be included in risk-of-bias assessments. We will follow the guidelines described by Vesterinen *et al*^33^ for conducting meta-analysis of data from animal studies. Meta-analyses will be conducted using the R statistical packages: dmetar V.1.0.0; metafor V.2.4-0. Normalised mean difference method will be used to calculate effect sizes as this approach can inform both the direction and magnitude of treatment effect relative to a normal, healthy control. If normative data are not reported, we will use standardised mean difference method instead. An effect size will be calculated for each comparison: a cohort of animals receiving treatment (ie, model induction or pharmacological intervention) versus a control cohort (ie, healthy and/or sham control and/or vehicle control). If a control group serves multiple treatment groups, we will give the ‘true number of control animals’ by adjusting for the number of treatment groups served. Each study will be weighted using the inverse variance method, where greater weight is given to more precise studies. We will use a random-effects model to combine effect sizes. The distribution of effect sizes has a weighted mean (the summary estimate), a weighted sum of the square of the deviations from that mean (the heterogeneity), and an estimate of the variance of the effect sizes beyond that expected by chance (tau-squared, τ^2^). We will use the restricted maximum likelihood estimate approach to estimate τ^2^. The presence of heterogeneity will be assessed by a combination of Cochran’s Q, I^2^ tests and relevance to the biological system (ie, differences in effect sizes between different species and sex). When the p value of Q is less than 0.05 (p<0.05), it indicates the presence of heterogeneity, whereas when p is more than 0.05 (p>0.05), it indicates the evidence of heterogeneity is lacking in the selected population. I^2^ gives information regarding the level of heterogeneity by calculating the proportion of total variance between studies that is due to true differences in effect sizes as opposed to chance. To interpret the I^2^ value, we will use the definition given by Higgins and Thompsons^34^: 0%–25% indicates very low heterogeneity; 25%–50% indicates low heterogeneity; 50%–75% indicates moderate heterogeneity; and >75% indicates high heterogeneity. To determine sources of heterogeneity, we will perform stratified meta-analysis according to the study characteristics which we think may be responsible for the observed heterogeneity. They are (1) animal species; (2) animal sex; (3) type of disease model (ie, the mechanistic nature of how disease model was induced); (4) type of intervention (ie, individual drug and drug classification base on the mechanism of action); (5) type of thigmotactic behavioural metric; and (6) items of study quality criteria. We will also perform multivariate meta-regression to assess the impact of other study-level variables on the effect size. The correlation between thigmotaxis and each type of limb withdrawal (ie, mechanical, thermal, cold) behaviour will be assessed using the Pearson correlation coefficient. Publication bias will be evaluated using (1) funnel plot, (2) Egger’s regression with a 95% CI and (3) non-parametric trim and fill. If publication bias is detected, we will then calculate a summary effect size to show the ‘true’ estimate of effect in the absence of publication bias.

## Discussion

The open field test is widely used, however thigmotaxis as a pain-associated outcome measure is still relatively novel although increasingly being employed. We anticipate that studies will differ in how the paradigm is performed and therefore it is important to understand the different factors that contribute to the variability we anticipate in the results. In order to gather all relevant studies as best as possible, we will manually screen through reference lists of the included studies to identify studies that are missed by the initial search. We are also confident that our approach to screening will ensure that we do not incorrectly exclude eligible and relevant studies. This review will inform the quality of studies by appraising the experimental rigour and evaluating their reporting transparency. It will also inform causative factors that are responsible for heterogeneity. The empirical evidence gained will improve study design, enhance methodological quality of experiments, stimulate reporting and research transparency of preclinical studies and contribute to applying the principle of the 3Rs (Replacement, Reduction, Refinement) in animal research. It should be noted that the review also has potential limitations. First, the included studies are likely to have high levels of variability in their experimental designs, conduct and reporting which may represent a limitation for the analyses. Second, the summary effect size may be overestimated due to the presence of publication bias. However, this could be addressed by using statistical methods to calculate a true estimate of the effect, filtering out the influence of publication bias.
